# Children's independent mobility during dark hours: a scoping review

**DOI:** 10.3389/fpubh.2023.1110224

**Published:** 2023-06-09

**Authors:** Anna Litsmark, Johan Rahm, Pimkamol Mattsson, Maria Johansson

**Affiliations:** Department of Architecture and Built Environment, Environmental Psychology, Faculty of Engineering, Lund University, Lund, Sweden

**Keywords:** children, independent mobility, after dark, darkness, outdoor lighting, street lighting

## Abstract

**Introduction:**

Independent mobility is every child's right and has implications for their health, wellbeing, and development. This scoping review addresses children's needs and experiences of light conditions in their everyday outdoor life. The review examines peer-reviewed scientific literature that analyses associations between different light conditions and children's independent mobility (CIM) during dark hours.

**Methods:**

By formulating a Boolean search string, including terms related to children independent mobility, light and outdoor environment, five scientific databases were searched. The search resulted in 67 eligible papers that were analyzed through an inductive, thematic analysis.

**Results:**

Four overarching themes representing the researched topics of the effects of light conditions with importance for CIM during dark hours were identified: (1) physical activity (PA) and active travel, (2) outdoor activities and place use, (3) safety perception, and (4) outdoor risks. The findings highlight that darkness constitutes a major obstacle for CIM, and that fear of darkness is common among children. It restricts the degree of CIM and influences children's safety perception as well as how they navigate through public places outdoors. The findings show that the type and design of outdoor settings during dark hours and children's familiarity with places during daytime could play a role in the degree of CIM after dark. The presence of outdoor lighting is related to children's increased PA and active travel, and outdoor lighting seems to also influence children's place use and interaction with the environment. The presence and extent of outdoor lighting and lighting quality may play a role in children's safety perception, which in turn can influence CIM.

**Discussion:**

The findings suggest that promoting CIM during dark hours might not only contribute to the accumulation of children's PA, confidence, and skills, but also support mental health. The understanding of children's perspectives on the quality of outdoor lighting needs to be deepened to support CIM. Highlighting the child perspective would aid the development of current recommendations for outdoor lighting and the implementation of the Agenda 2030 of ensuring healthy lives and promoting wellbeing for all at all ages, and making cities inclusive, safe, resilient and sustainable throughout the day and seasons.

## 1. Introduction

The United Nations Agenda 2030 states that cities should provide access to safe, affordable, accessible and sustainable transport systems to all citizens, including children (1). Children's independent mobility (CIM), i.e., the degree to which children of different ages have the freedom for independent action, exploration, play and socializing with friends in their local environments without adult supervision (2), has an intrinsic value for children and is something that they have the right to enjoy (3). This right is formulated in the United Nations' Convention on the Rights of the Child, enshrining that every child has the right to rest and leisure, to engage in play and recreational activities appropriate to the age of the child (Article 31) and to a standard of living that is good enough to meet their physical and social needs and support their development (Article 27) (4). A prerequisite for this is a safe outdoor environment (3).

Light and darkness affect how places are used and perceived (5, 6). It influences our behavior, such as how we interact with others, position ourselves and navigate through public places (7–9). Well-lit outdoor environments are, among adults, associated with perceived visual accessibility, safety, and walking (10). Little research has focused on children's needs and experiences of light and darkness in their everyday outdoor life, implying that lighting recommendations and standards are informed by research based on adults' perceptions and needs (11).

In the history of studying CIM, the permission to go out after dark has been included in measures of mobility licenses that children obtain from their parents. In the seminal study by Hillman et al. (12), the question “Is your child usually allowed to go out alone after dark?” was asked. Today, this question is one of six core questions typically included in questionnaires used to assess CIM (13). Yet, constrains and facilitators of CIM during dark hours seem to be an overlooked topic. A study of CIM in 16 countries^1^ across the world consistently showed that darkness constitutes a significant barrier to CIM (3). Going out alone after dark was the most withheld independent mobility (IM), and only 22% of the children were granted permission by their parents to go out alone after dark. The authors recommended “Single Double Summertime”^2^ resulting in lighter evenings to support CIM and reduce road causalities, but neither potential benefits of CIM during dark hours nor outdoor lighting to support CIM was discussed.

Though CIM during daylight has received considerable attention [e.g., Marzi and Reimers (13), Malone (14), Schoeppe et al. (15)], less focus has been placed on the dark hours and how artificial outdoor lighting may support children's needs for IM in their neighborhood. The impetus for exploring this issue is the past 40 years research showing that CIM is declining, with significant implications for children's health and physical, social and mental development (3). This also applies to the Nordic countries (16–18), where the dark season constitutes a particular challenge for children as it entails extended hours of darkness. Children's perceptions of place differ from adults, highlighting the necessity for research focused on children's perspectives (19). To counter the decline in CIM, there is a need to peer into the darkness to fully understand the complex interdependencies between qualities of urban environments, parental concerns and CIM (20–22).

### 1.1. Aim

This scoping review aims to identify and map the available scientific knowledge about CIM during dark hours and further identify knowledge gaps for future studies. We investigate differences of CIM between natural light and darkness. Furthermore, we consider how artificial outdoor lighting may support CIM on foot or by bicycle within neighborhoods during dark hours. The review is based on three overarching research questions:

RQ 1: How and under which circumstances has CIM during dark hours been studied up until now?

RQ 2: What are the effects of light conditions on CIM, and for whom and where are the effects reported?

RQ 3: How are the light conditions defined and operationalized in relation to CIM in previous studies?

The overall goal with the review is to provide knowledge that could make cities more accessible for children throughout the day and seasons.

## 2. Method

### 2.1. The search protocol

The design of the review procedure was based on literature on scoping reviews (23, 24) and on the Preferred Reporting Items for Systematic Reviews and Meta-Analyses (PRISMA) statement (25). The procedure was initially defined by a review protocol expressing the purpose, search terms and eligibility criteria. The purpose of the review was expressed as follows: to identify, analyze, and describe the available research on children's perspectives, experiences, behaviors and responses to light condition. Search terms were defined in four groups to facilitate the development of a Boolean search string (1) related to the individual (e.g., child, pupil), (2) the activity (e.g., mobility, travel), (3) light conditions (e.g., street lighting, dark^*^) and (4) the setting (e.g., outdoor^*^, neighborhood). To avoid the scope being too narrow, both variations in natural light and artificial outdoor lighting were considered. After initial searches that combined the search terms in different ways, a final Boolean search string was created. The protocol stated that to be included in the review, papers had to (a) be published in a peer-reviewed scientific journal, (b) be retrievable from internationally available electronic databases, (c) be written in English, (d) include an empirical study regarding children within school age (from the age of six up to 18 years old), (e) focus on urban outdoor settings and (f) include light conditions in the result section. No exclusion criteria were applied regarding date of publication or geographical origin.

### 2.2. The search process

**Figure 1:**
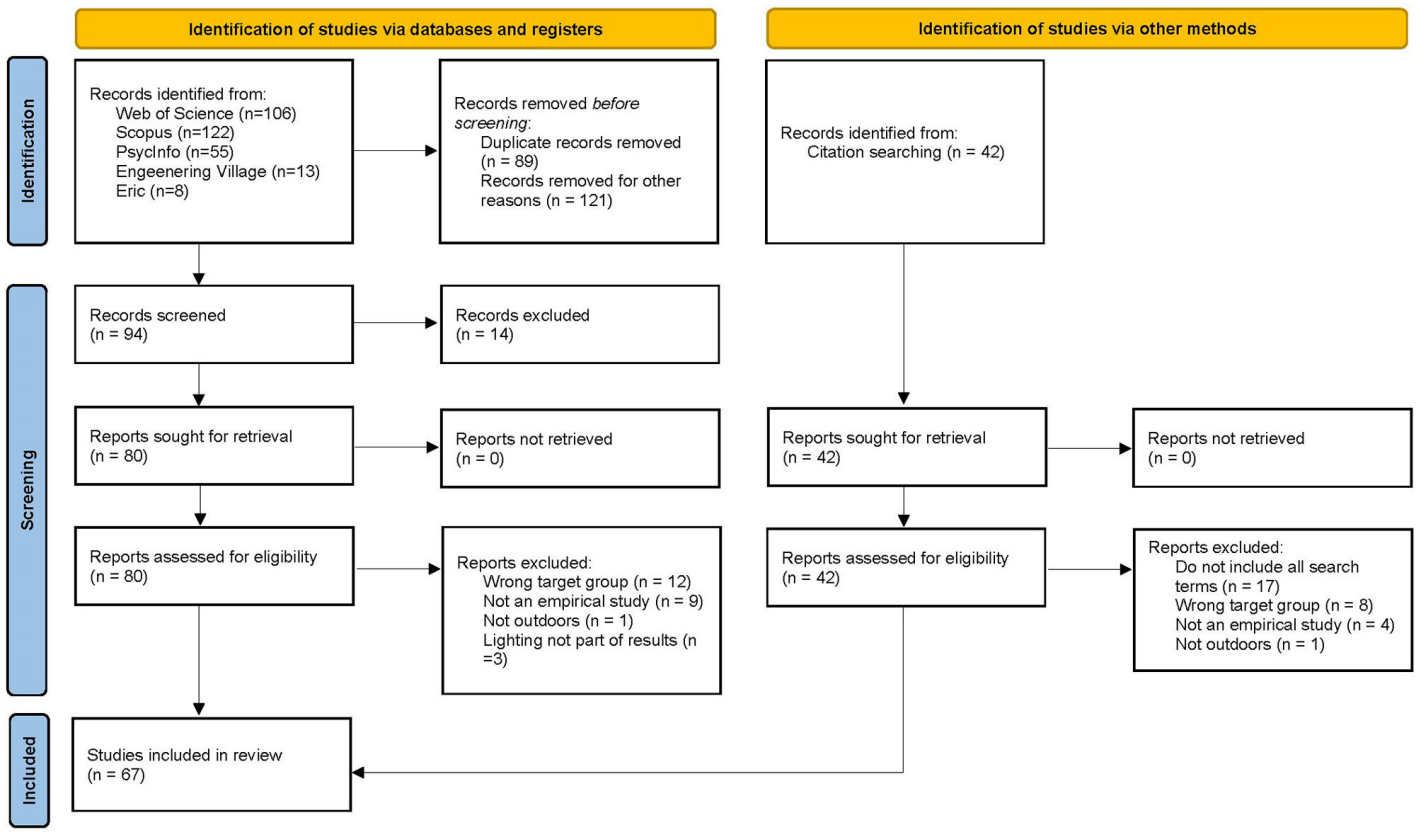
PRISMA 2020 flow diagram for the review. Figure adapted from Page et al. (25).

In order to cover a wide range of disciplines, the search was conducted within several electronic databases. To get a second opinion on the search strategies and choice of databases before initiating the searches, Lund University library staff was consulted. The searches were then conducted in the following databases: Scopus, Web of Science (ISI), PsycInfo, Eric and Engineering Village up until April 2021. The search string functioned as a template and was adapted to the different databases (See Supplementary Table 1). Relevant papers were identified by the title and, if needed, by reading the abstract. The search resulted in 304 relevant hits out of a total of 3,348. Eighty-nine duplicates were eliminated, and 121 papers were excluded because they did not include all four groups of the search terms. The remaining 94 papers were screened in detail by reading the abstract and, if necessary, screening the full-text to assure that the search terms were fulfilled. Fourteen of the papers were excluded because the search hits referred to something other than what was intended (e.g., *light* physical activity instead of artificial light). The 80 papers were read by four researchers who independently screened whether the abstracts in addition to the inclusion criteria contained all four aspects of the search terms: individual, activity, light conditions and outdoor environment (setting). The researchers agreed that 55 of the 80 papers were relevant for the present study. The remaining papers' full-text versions were retrieved and read. Twelve additional papers were identified in the reference lists of the selected papers. These were retrieved and assessed for eligibility by full-text reading. The final number of papers eligible for further analysis were 67 (See Figure 1).

To capture the intended scope of RQ 1, a descriptive analysis of the included papers' origin and methodological standpoints was made. The analysis focused on year of publication, type of journal, country, and continent of origin, how CIM was defined and studied, whose perspective the findings were based on, the age of the studied children, in which setting the studies has been carried out, how the light conditions were defined, and methods and theories used.

To answer RQ 2 and RQ 3, the papers were then analyzed with an inductive, thematic approach inspired by Braun and Clarke (26). First, the papers were read, and initial ideas were noted down (step 1). Then, initial codes based on the issues of CIM they explored in relation to light conditions were generated (step 2). Thereafter, the coded papers were organized into potential themes (step 3). The themes were reviewed by checking if they worked in relation to the codes generated in step 2 and if they were representative of the entire set of papers collated in step 3. Categories defining the studied light conditions were then identified within each theme. Lastly, themes and categories were defined and named.

## 3. Results

### 3.1. How and under which circumstances CIM during dark hours has been studied

**Figure 2:**
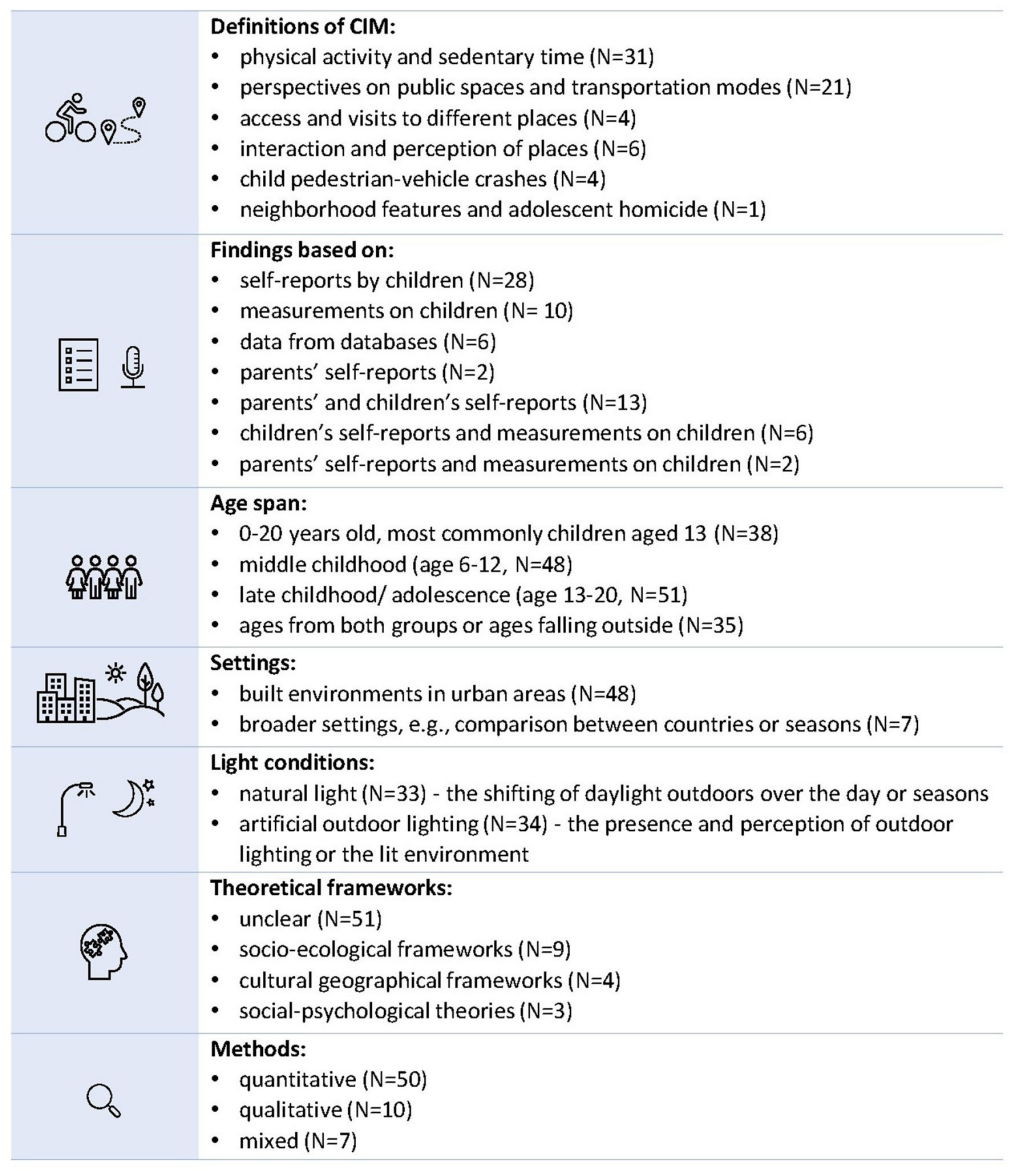
Summary of how CIM during dark hours has been studied.

The 67 identified papers, dated from 1999 to 2021, were published in 45 different journals, primarily within the fields of public health, medicine, and transportation research. Most of the papers were based on research in Europe (*N* = 26) and North America (*N* = 21). The remaining papers were based on research in Oceania (*N* = 12), Asia (*N* = 3), South America (*N* = 2) and Africa (*N* = 1). Two papers included data from several continents. In the following sections, it is described how CIM was defined and studied in the identified papers, whose perspective the findings were based on, the age of the studied children, in which setting the studies were carried out, how the light conditions were defined, and the methods and theories used (see Figure 2 for a summary and Supplementary Table 2 for further details).

CIM has been defined and studied in different ways in the papers. About half of them (*N* = 31) studied children's physical activity (PA) and sedentary time in relation to environmental features, e.g., season, day length or dark streets, or in relation to the perception of an area, e.g., perceived safety. Twenty-one of the papers studied children's or parents' perspectives on public spaces and transportation modes to different destinations and explicitly expressed a focus on children's everyday IM. Other papers are more implicit in their definition of CIM but studied the access and visits to different places (*N* = 4), or the interaction and perception of places from a broader perspective (e.g., in relation to transitioning toward adulthood or place connectivity) (*N* = 6). A small number of the papers (*N* = 4) focused on child pedestrian-vehicle crashes during different environmental conditions (e.g., after sunset). One paper studied the associations between environmental neighborhood features and adolescent homicide.

Most of the papers based their findings on self-reports by children, e.g., through questionnaires, interviews or focus group discussions (*N* = 28), or on measurements on children (e.g., daily step counts (DSC), heartrate (HR), global positioning systems (GPS) monitoring) (*N* = 10). Six papers based their findings on data from databases (e.g., data on pedestrian-vehicle crashes, adolescent-homicides, physical activity). Few were based on parents' self-reports, e.g., through questionnaires or interviews (*N* = 2). Other papers based their findings on both parents' and children's self-reports (*N* = 13), children's self-reports and measurements (DSC, HR, GPS monitoring or body mass index (BMI) (*N* = 6) or parents' self-reports and measurements on children (BMI) (*N* = 2).

The children in the papers were 0–20 years old, where the most common age group was children aged 13 (*N* = 38). Overarchingly, the children could be divided into two age-groups considering this review's focus on school-aged children: middle childhood (age 6–12, *N* = 48) and late childhood/adolescence (age 13–18, *N* = 51). Thirty-five papers included ages from both groups or children younger than six and/or older than eighteen. This means that some of the children's ages fall outside the specified age in the eligibility criteria, but also that some of the children were older than what is defined as a child according to the United Nations Convention on the Rights of the Child (i.e., every human being below the age of eighteen years) (4).

The studied settings ranged from children's local neighborhoods to a focus on e.g., park features or road environment design. Most common were built environments situated in urban areas, e.g., public open spaces, road environments, recreational facilities, parks, the way to school or activity spaces for children (*N* = 48). Some papers focused on a broader setting (e.g., made comparisons between countries or seasons) (*N* = 7).

The light conditions considered in the papers were described in a variety of terms (see Supplementary Table 2 for details), but can be divided into two major groups: (1) natural light (*N* = 33), including the shifting of daylight outdoors over the day or seasons, e.g., evening and night-time hours, day length differences over the year, after dark or darkness, and (2) artificial outdoor lighting (*N* = 34), comprising studies of outdoor lighting and the lit environment, e.g., the presence of outdoor lighting in an environment or the perception of outdoor lighting or the lit environment. Hereafter we will use the terminology *natural light* and *outdoor lighting* to distinguish between the two groups and use *light conditions* as an overarching concept to describe both.

Considering how the relationships between children and the environments were framed, most papers were unclear in their theoretical standpoint when studying associations between CIM and characteristics in the physical environment (*N* = 51). A small number of the papers explicitly applied a socio-ecological framework considering the interplay between children and their physical and/or sociocultural environments (*N* = 9) [e.g., Sallis et al. (27), Lang and Rayner (28)]. Some papers relied on cultural geographical frameworks [e.g., Holloway and Valentine (29), Kato (30), Vanderstede (31)], looking into the cultural dimensions of space and place of adolescents' everyday life (*N* = 4), while three papers applied social-psychological theories (32–35) to explore parents' attitudes and beliefs toward active school travel, outdoor play or to understand associations between environmental features and adolescent homicide. Quantitative methods and analyses (*N* = 50) were most common for studying associations between child behavior and environmental factors. Ten papers used a qualitative methodology and seven used mixed-method approaches (*N* = 7).

### 3.2. Operationalization of light conditions and effects on CIM

**Figure 3:**
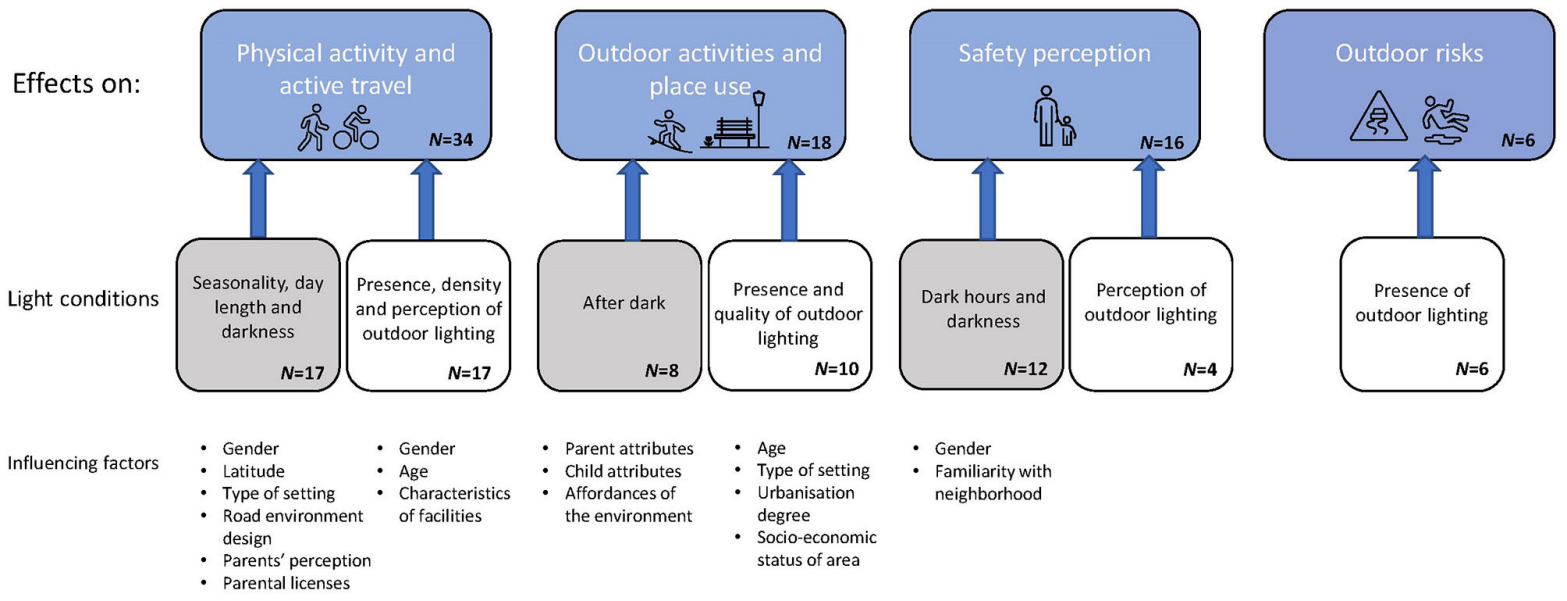
Identified themes that light conditions have an effect on. Some papers are included in more than one theme. Light conditions constitute identified categories and their main characteristics. Gray boxes regard findings in relation to natural light and white boxes regard artificial outdoor lighting. Influencing factors includes identified key aspects connected to each theme and category.

The thematic analysis generated four overarching themes related to the effects of light conditions on CIM: (1) *physical activity and active travel*, (2) *outdoor activities and place use*, (3) *safety perception* and (4) *outdoor risks*. The first three themes were further divided into two categories, related to the identification of effects of (1) variation in *natural light* or (2) presence of artificial *outdoor lighting*, focusing on e.g., quality. The fourth theme only included results regarding artificial lighting. For some categories, influencing factors were identified. These factors are indicated in italics in the text and constitute key aspects in relation to physical or social environmental factors, influencing the effects of light conditions on CIM. The themes, categories and influencing factors are outlined in Figure 3.

#### 3.2.1. Physical activity and active travel

The first theme, including 34 papers, regards how light conditions influence children's physical activity (PA) (e.g., daily steps) and active travel (traveling by bike or by foot), and is divided into two different categories with regard to the studied light conditions: one focusing on natural light including seasonality, day length and darkness and the other focusing on artificial outdoor lighting, including the presence, density and perception of outdoor lighting. In both categories, children's behaviors primarily were studied through measurements of daily step counts, heart rate, BMI, or GPS, while in some papers, the behaviors were captured through self-reports. Children's perceptions of outdoor lighting were investigated, and some papers also captured children's perspectives on outdoor lighting for supporting their outdoor PA.

##### 3.2.1.1. Natural light: seasonality, day length and darkness

Seventeen papers considered natural light in relation to children's PA and active travel. The findings are somewhat inconsistent, but most findings suggest that darkness with regard to day length and seasonality is a barrier to children's PA and active travel. Children seem to be more physically active when the day length is longer, i.e., during summer months.

Five of the papers have found associations between seasonality and children's PA (36–39) or active travel (40). In three of the papers, there are differences depending on *gender* and *latitude*. Among European children (age 12–17), a stronger relationship between seasonality, PA and sedentary time were found among children in Central-North of Europe compared to South of Europe (37). Associations were only significant for girls' sedentary time. More extreme winter conditions (i.e., darker and colder) were suggested as an explanation for the geographical differences in activity/sedentary behaviors. In the US, there was a clear seasonal pattern of children's active trips, with high levels during summer months and low levels during winter months (age 5–17). Also, children were more sensitive to seasonality than adults and older adults (40).

Six papers found associations between different months, hours of daylight, day length or exposure to natural light and children's PA (41–46) relating to gender and latitude. Children (age 9–13) living in the UK engaged in significantly less PA minutes during winter/spring months compared to autumn months (45). Among children (age 3–18) from Australia, Europe and the US, more daylight hours and better visibility were related to increased PA (43). Day length was associated with children's PA, but the association varied between the included countries. One Southern European country (Portugal) had an opposite trend, i.e., decreased hours of daylight was associated with increased PA (43). Children in Northern Europe and Australia remained more active given the weather conditions than children in the US and Western Europe (43). In the US, each additional hour of day length was associated with more PA among children (age 12–14) (44). On days with 14 h of daylight or more, British children's PA (age 8–11) was higher, but there was no difference between short and medium days (< 9.5 h/10.2–12.6 h) (46). The effect of long day length was largest between 5 p.m. and 8 p.m. and remained so after adjusting for the effects of different weather (e.g., rainfall, wind). This effect was explained by greater duration and intensity of play outside home on long days. Active travel and structured sports were less influenced by the day length (46). Among children (age 5–16) in Australia, Brazil, Europe and the US, longer evening daylight was independently related to a small increase in daily PA, with largest associations in the late afternoon and early evenings (41). However, these associations were inconsistent, with stronger associations between day length and PA among boys in some countries (i.e., Denmark and England) (41). In another paper, British children's (age 5–15) average daily light exposure was positively associated with time physically active and negatively associated with sedentary time (42). The associations seemed to have an independent nature, suggesting that exposure to light may be associated with PA and sedentary behavior separately, thus not simply displacing each other.

In three papers, *urban and rural areas* were studied in relation to associations between season, PA and active travel (36, 38, 39). Children (age 11–12) from both urban and rural areas in Cyprus spent significantly more time active outside during summer than winter (36). Compared to the children in urban schools, children in rural schools spent significantly more time outside over the two seasons (36). Interestingly, urban school children were more active in the winter and rural school children were more active in the summer. In Scotland, levels of PA were highest among both urban and rural children (age 10–11) during the summer months, and lowest in the autumn months (39). In Canada, children (age 9–10) reported lower PA during winter (38). Winter months had lower odds of children engaging in active school travel compared to the fall, and were greater among boys compared to girls, and children living in urban vs. rural areas.

Associations between evenings or hours after dark, PA and active travel have been found regarding *gender, road environment design, parents' perception* and *parental licenses* (47–51). Beyond daylight hours, few girls (age 15) were physically active outside (51). Several of them wanted to keep their recreational activities after dark, but were constrained by fear, parental concerns, lack of supervision and suitable transport. During evenings, boys' (age 13–15) PA were positively associated with the presence of speed humps (47). However, Carver et al. (52) found no clear difference in children's (age 8–9, 13–15) PA before school, after school or in the evening. Comparisons between parental licenses and active transport by sex revealed that boys (age 12–14) were 1.9 times more likely than girls to be allowed out after dark in New Zeeland (49). In Sweden, parents drove their children (age 7–13) to school by car during the winter, due to cold weather and bad conditions for cycling (50). Good outdoor lighting was perceived by parents as important to enable children's travel to school during the long period of darkness that the winter entails (50). When parents viewed their neighborhood as unsafe for their children (age 3–10) to walk after dark, the children were more likely to be obese (48).

##### 3.2.1.2. Outdoor lighting: presence, density, and perception of outdoor lighting

Seventeen of the papers within the theme studied outdoor lighting in relation to children's PA and active travel. The papers show that outdoor lighting has an influence on children's PA and active travel, but that it varies depending on factors such as *gender, age*, and *characteristics of facilities*.

In papers where the influence of presence or density of outdoor lighting on PA has been studied, the findings are contradicting. Two papers showed that the presence of outdoor lighting was related to increased PA among boys (age 10–14) (53) and active travel for both boys and girls (age 6–16) (54). Papers also showed that a higher density of outdoor lighting was positively associated with children's objectively measured PA (age 16–20) and with increased self-reported walking (age 0–18) (55), and that children (age 11–12) living in areas with more outdoor lighting engaged in more PA (56).

Two papers found no association between the presence of outdoor lighting and PA among children (age 9–14) of both genders (57, 58). Among boys (age 8–9), public spaces with no lighting along paths were inversely associated with PA during weekends (59). Even though presence of outdoor lighting could be viewed as a safety feature, other *characteristics of facilities* in public places might matter as well (59). The places with outdoor lighting could, for example, be “trouble spots” or formal recreation places, thus being spaces that the boys are not allowed to visit or that they are not interested in visiting (59).

Ten papers focused on children's perception of outdoor lighting and how it influences PA and active travel (60–69). The findings indicate that children's perception of well-lit environments support PA and active travel. Having no outdoor lighting on the street were seen as a physical environmental barrier among children (age 8–13) from underserved neighborhoods, and dark streets were mentioned as barriers to their ability to be physically active (65). Children (age 12 and 18) agreed somewhat with a statement that poor outdoor lighting was a constraint on the choice to walk (67).

Eight of these papers highlighted effects of the perception of outdoor lighting and PA or active travel with regard to *gender*. For both genders (age 15–16), the perception of well-lit streets was positively correlated with active commuting to school (63) and the convergent validity for children's (age 10–12) transportation to school was fair regarding the statement “The route does not have good lighting” (68). Sufficient outdoor lighting was perceived as a PA-facilitator among children (age 10–11) of both genders (66). For girls, the perception of good outdoor lighting at night were found to predict active transport (age 15–17) (64) and higher non-school PA (age 11–12) (60). Additionally, girls' (age 15–17) perception of poor outdoor lighting was associated with 40% greater probability of not engaging in PA in urban parks (61) and it has been suggested that environments with better outdoor lighting could increase girls' (age 15 and 18) PA (69). On the contrary, girls' (age 13–14) perception of their neighborhood as being well-lit was associated with steeper declines in non-school PA (62). *Age differences* could be a reason why, e.g., well-lit streets might serve as an important venue for non-school activity for girls 11–12 years old, but not for girls 13–14 years old. Another reason given by the authors was that reporting that streets in one's neighborhood are well-lit cannot be interpreted as girls being out at night (62).

#### 3.2.2. Outdoor activities and place use

The second theme, including 18 papers, focuses on the influence of light conditions on children's outdoor activities (e.g., play, being with friends, route choices), place use, interactions with their surroundings and identity development. Children's views and experiences are captured to a various extent, with some of the papers basing their findings on parent's self-reports, while others are based on self-reports by children. The theme is divided into two categories with regard to natural light and artificial outdoor lighting: one focusing on the constraints of darkness on children's outdoor activities and the other on children's place use and identity development in relation to presence and quality of outdoor lighting.

##### 3.2.2.1. Natural light: after dark

Eight of the papers reported how darkness has a great influence on children's possibilities to be outdoors. According to findings, darkness restricted children from being outdoors by influencing parent practices, was associated with children being indoors, and was a signal that it is time to go home for both children and parents. However, darkness was not always associated with restrictions.

Three papers showed that *parent practices* were impacted by darkness and that CIM was restricted after dark (49, 51, 70). In New Zeeland, there was a drastic reduction of children (age 12–14) being permitted out after dark between generations (49). Almost 30% of the parents were permitted out after dark when they were children, while only 15% of the children today were permitted to go out after dark. Children (age 12–14) were subject to *parental constraints* relating to walking alone when it was dark and perceived “no-go” areas which they avoided (70). In line with this, only 31% of girls (age 15) were allowed to be in parks after dark by their parents (51).

Three papers showed that darkness was *associated with being indoors* and a signal that it is time to go home for both children and parents (71–73). Summer was described by children (age 8–10) as a “big, long play time” which only ends when it gets dark, while in winter many retreated from weather and shorter daylight hours to play indoors (71). The weather, shorter days and *limitations of affordances* were seen as barriers to the use of public open spaces in winter (72). Only the families that perceived values in outdoor play during winter had children that felt the urge to play outside (71). Hours of daylight also seemed to influence how children (age 0–15) allocated their time across different activities (73). When daylight durations were longer, children allocated more time to outdoor activities during weekends and spent more time on school-related activities during weekdays.

Two papers indicated that there existed *exceptions of restrictions* of CIM (age 14–16) after dark in Spain (74, 75). Children's possibilities to be outdoors after dark could be encouraged at particular events, such as at neighborhood festivals. Parents let their children stay out longer than usual since there were more people around, thereby being viewed as safer for their children. The festivals were moments of night-time leisure when both parents and their friends and neighbors were outdoors late.

##### 3.2.2.2. Outdoor lighting: presence and quality of outdoor lighting

Ten of the papers described how outdoor lighting may influence children's place use, but also play a role in their interactions with their surroundings and identity development. The findings indicated that positive effects of outdoor lighting on CIM varies *depending on age* and *where the child lives*.

The presence of outdoor lighting seemed to influence park use among children of different ages (76, 77) and good outdoor lighting was viewed by children (age 13–17) as one of the most important physical characteristics of an activity-friendly environment (78). Parks which children aged 6–8 years old visited had significantly higher chance of having outdoor lighting compared to parks that children aged 3–5 and 9–11 years old visited (77). Lighting around courts was associated with children aged 12–15 years old's park use (76). However, presence of outdoor lighting seemed not to influence children's cycling route choices. Children (age 13–15) chose the shortest possible cycling routes over routes that were covered by lighting (79). In addition, the routes chosen by children (8–12) had less outdoor lighting compared to the shortest routes (80). It is unclear what influenced these children's route choices. Dessing et al. (80) reflected that most of their findings were based on data collected around spring and the beginning of summer, which could have influenced the children's transportation behavior. During dark winter morning trips, outdoor lighting might play a more crucial role in children's walking and cycling route choices. Similar matters were discussed by Verhoeven et al. (79). Bad weather and fewer hours of daylight may impact both children' route choices and transportation mode, even if their findings do not show so. Their data was collected during autumn and winter, but the time for sunrise and sunset was not defined, meaning that it is difficult to get an understanding of the actual light conditions at the studied times.

Outdoor lighting could affect *older children's* interaction with places and support or hinder their sense of identity, belonging, and transition toward adulthood (81). Darkness disrupted children's (age 14–15) micro-sociality of spaces and both outdoor lighting and darkness affected their interactions with their surroundings. It was proposed that children create affective mental maps that guide them where to go and not go depending on light and darkness (81). The night-time was viewed as a forbidden sphere for children while having a strong symbolic value that offers them (age 14–16) the possibility to avoid parental control and develop relationships with peers (74, 75). Public spaces at night could be influential in the creation of youth identity (82) and open up for new possibilities and several “firsts,” which could be important experiences in the liminal age that adolescence is. In familiar spaces, such as a child's neighborhood, children could acquire social and spatial skills that are crucial when navigating spaces by themselves (75).

There seemed to be different possibilities for children to be outdoors after dark depending on *urbanization degree* and *socio-economic status* (81, 83, 84). Urbanization led to a decrease of children's (age 10–18) licenses to independently spend time with friends outside after dark (84). Public open spaces in neighborhoods with higher socioeconomic status were more likely to have features supporting place use (e.g., outdoor lighting), compared to public open spaces in lower socioeconomic neighborhoods (83). Children (age 14–16) experienced dimmed, broken, or absent outdoor lighting as a material manifestation of neglect from powerful institutions and class devaluation (81).

#### 3.2.3. Safety perception

The third theme, consisting of 16 papers, highlights how light conditions can influence children's perception of safety in different environments. Unlike above themes, this focuses to a greater extent on children's views and perceptions of light conditions and how it influence everyday outdoor life, including CIM. One category regards natural light focusing on dark hours and darkness, and a second on outdoor lighting with a focus on the presence and quality of outdoor lighting.

##### 3.2.3.1. Natural light: dark hours and darkness

Twelve of the papers had findings related to natural light and children's perception of safety. The papers highlighted that many children were afraid of the dark and that darkness influenced their perception of safety in urban environments. Further, darkness constituted a barrier to CIM in terms of going out after dark and places to be avoided. The perception of fear and safety and coping strategies and avoidance behavior to handle/avoid negative feelings varied between the papers.

Ten papers highlighted *gender differences* for CIM after dark. Gender differences were significant for fear of darkness among children between 11 and 16 years old, with more girls reporting such fear (85, 86). Fear was reported more often by 13-year-old children who believed that their parent's opinion was that children of their age should not be walking in town or riding a bicycle in the evening (86). Girls (age 13–16), regardless of the time of day, felt less safe in public spaces than boys did (87). Boys were more likely than girls to indicate that their local areas were a safe place to walk alone after dark and felt generally safer all the time in their local area compared to girls (85). Children of both genders reporting more traffic and/or car parking on their local streets were less likely to perceive their neighborhood as a safe place to walk alone after dark (85) thereby, suggesting the relation between the number of vehicles and perceived walkability among the children. The fear of darkness also varied among children (age 13–14), depending on factors such as *housing type, family characteristics* and *parental licensing* (88). Among girls, mobility was more a reflection of their feelings when moving in their neighborhood, while among boys, housing conditions and the *traffic environment* seemed to be crucial factors influencing their IM (88). For both genders, children's (age 10–18) feelings of safety were lower when traveling on foot than traveling by car or public transport (89). Pursuing activities at night (e.g., eating, shopping, and hanging out) seemed also less safe to children (age 14–18) than during day, and pursuing an activity during the night decreased the perception of safety at the location of the activity, particularly among girls (90). By both genders, the most frequently mentioned reasons for perception of urban threats were a fear of (1) darkness and lack of outdoor lighting, (2) people in general, and (3) locations with negative associations (e.g., cemeteries and dark underpasses). The fear of the dark was probably the most serious gender difference in the perception of walkability (87).

*The location of CIM* after dark seemed as well to be affected by gender. Girls (age 13–16) perceived larger areas to be dangerous during the night more than boys did (91). Regardless of the time of the day, the boys' collective walking activity space was also distributed over a larger area compared to that of girls. The biggest difference was found after dark, where girls' distribution of spaces was 4.11 km^2^, while boys' distribution reached 6.86 km^2^ (91). Moreover, different spatial patterns for children's (age 13–16) location of perception of safety was identified (87). Cemeteries, parks, train stations, side alleys and places with pubs were bound to the greatest perception of fear during the night. Girls stated that they were more afraid in parks mainly due to the dark and insufficient outdoor lighting, lack of people, and threats from passers-by, while cemeteries were more often mentioned by boys (87). Children of both genders (age 10–18) felt less safe in areas where alcohol outlets were overly common (89) and said that dark, lonely places should be avoided (age 15–16) (92). Reasons stated were social threats such as “rapists” and inebriated adults. Among girls, safety strategies such as avoiding being alone in certain places after dark or acting confident (i.e., not scared) when being alone in public spaces after dark were mentioned (92). During daytime, almost all children (age 8–9 and 13–15) believed that it was safe to walk around the block alone, while almost half of them considered it safe to walk home from a bus or train stop at night (93). Among girls, an increased level of concern about road safety impacted their outdoor activities negatively during evenings (93). There were few places outside their homes in which girls (age 15) felt safe after dark (51). A majority of the girls did not perceive natural outdoor environments as safe during dark hours. Surprisingly, however, some of the girls described that if they would feel safe, recreation under the cover of darkness would be beneficial, as darkness was seen as psychologically safe and as a condition under which they could avoid the critical gaze of others (51).

Two papers highlighted a different perspective on safety perception after dark, by mentioning the *familiarity of the location* (75, 94). Familiar neighborhoods providing feelings of safety and confidence supported the conditions for an autonomous experience among children (age 14–16) (75). The neighborhood was described as the primary space where adolescents developed their leisure practices during both day and evening and the spatial knowledge acquired during daytime played an important role in the discovery of nightlife (75). In suburban and rural locations, familiar home areas were seen as less threatening. Compared to urban areas, lone travel after dark was more common among children in these locations (age 13–14) (94).

##### 3.2.3.2. Outdoor lighting: perception of outdoor lighting

There are only four of the papers within this theme with findings related to outdoor lighting and children's perception of safety. All stressed that the presence and quality of outdoor lighting in an area can play a role in children's perception of safety, which in turn can influence CIM.

Outdoor lighting at night was found to be positively associated with perceived safety among children (age 8–10) (95, 96). Based on color information extracted from an image captured from the International Space Station (ISS), it was suggested that children who lived in neighborhoods with greater green or blue hued (compared to red) outdoor lighting at night felt safer and that children felt less safe if they lived in neighborhoods measured to have low-medium compared to medium amount of outdoor lighting (96).

Two papers studied more in-depth children's views on outdoor lighting in relation to perceived safety. An area was described as safe due to the fact that it was perceived as well-lit (age 14–18) (90) and children (age 14–15) explained how inadequate outdoor lighting was an issue with regard to their perception of safety (81). Poorly lit spaces were avoided when it was dark, and evoked fear due to obstructed visibility and recognition. For some of the children, darkness signified danger and poor lighting made them feel anxious or scared. Therefore, they requested more, or brighter outdoor lighting as a way to feel safer (81).

#### 3.2.4. Outdoor risks

The fourth theme, including six papers, focuses on the risks of child injuries and death in urban environments under different light conditions. The papers in this theme predominantly studied risks based on accident reports, hospital discharge or death certificate data, i.e., looking at number of incidents in relation to light conditions in the built environment. One paper included children's views on risks related to absence of outdoor lighting and active school travel. The theme includes only one category focusing on the presence of outdoor lighting.

##### 3.2.4.1. Outdoor lighting: presence of outdoor lighting

Presence of outdoor lighting in an urban environment had an influence on the number of accidents involving children (97–100). Most child pedestrian injuries (age 0–18) seemed to take place at times of optimal conditions for driving (good lighting, dry road, good weather), indicating that optimal driving conditions likely represented optimal play conditions—thereby increasing the exposure of children to traffic (99). In this paper, which studied a total number of 3,823 vehicle crashes involving children, more than three quarters occurred in daylight, 16% of the incidents happened after dark in the presence of outdoor lighting, and < 1% in darkness. Spring months (April to June) had the highest occurrence of child pedestrian crashes and January had the lowest. Half of the injuries occurred in the late afternoon/evening (99). Similar diurnal and seasonal patterns were reported by others (97, 98). In the paper by DiMaggio and Durkin (98), the incidences were highest (i.e., 29.3%) for children aged 15–19 years old. For any age group, dark unlighted roads played little role for the risk of injuries. However, children (age 0–15) who were involved in crashes after sunset had a greater risk for more severe injuries or death (100).

Outdoor lighting was significantly associated with decreased risk of homicide among children (age 13–20) (101). The findings were explained by the broken windows theory, suggesting that neglected environments create more disorder and crime, or that outdoor lighting promote increased pedestrian activity and community interaction, i.e., promoting social connections and facilitating social control—thus reducing crime (101).

Only one paper highlighted children's perspectives on risks and injury and the parents' role for CIM in a post disaster community. Inoperative lighting along the way to school led to minor injuries among children (age 1–18), as they could not detect potholes in the road surface, which in turn induced fear among the children and caused parents to drive them to school during dark hours (102).

## 4. Discussion

This scoping review aims to identify and map the available scientific knowledge about CIM during dark hours by investigating how outdoor lighting may support CIM. Our definition of CIM has been comprehensive, referring to children's freedom for independent action, exploration, play and socializing with friends in their local environments without adult supervision (2), which agrees with the broad definition and interpretation of CIM in current literature (13). The internationally available, peer-reviewed scientific research identified, strengthens the perspective that darkness constitutes a major obstacle for CIM, and reveals physical activity (PA) and active travel, outdoor activities and place use, safety perception, and outdoor risks, as major research topics of the effects of light conditions with importance for CIM during dark hours. There are, however, an unevenness between the researched topics; most papers concerned effects on PA and active travel, while only a small number addressed effects on outdoor risks. This review also sets the effects of natural light and artificial outdoor lighting in relation to child characteristics and local contexts, likely influencing the impact of light conditions on CIM. The findings point to the need to deepen the understanding of children's perspectives on the quality of outdoor lighting to support CIM during dark hours among both boys and girls of different ages.

In the coming sections, we connect the findings to our research questions, discuss them in relation to previous research, especially stressing aspects that should be addressed in further studies and pointing to specific knowledge gaps. In the final part we critically reflect on our search method and summarize our conclusions.

### 4.1. Identified effects of CIM during dark hours and knowledge gaps for future studies

In response to RQ 1: *How and under which circumstances has CIM during dark hours been studied up until now?* and RQ 2: *What are the effects of light conditions on CIM, and for whom and where are the effects reported?*, we highlight the findings in relation to light conditions within the two major categories: *natural light* and artificial *outdoor lighting*. In the identified papers, much focus is put on children's PA, active travel, route choices, transportation mode, fear, safety perception and possibilities to be outdoors, whereas some aspects of CIM, in relation to darkness and outdoor lighting (such as exploration, play and socializing), are only mentioned to a limited extent. Hence, such essential aspects of children's lives are overlooked (103–105) with regard to darkness and lighting outdoors, making it difficult to meet the needs of children's right to rest, leisure and play in accordance with the Convention on the Rights of the Child (4). Despite this, the findings illustrated how natural light and outdoor lighting can influence CIM in different ways.

Natural light has an influence on children's PA and active travel. Darkness, with regard to day length and seasonality, comes through as a barrier to children's PA. Children of all ages seem to be more physically active at times when the day length is longer, i.e., during summer months. However, local circumstances in the built environment seem to be a deciding factor when it comes to how light and darkness influence children's PA and active travel. Environmental factors, such as degree of urbanization, the design of the road environment, and factors such as the latitude of the country could play a role. It is widely acknowledged that significant changes in the built environment and urbanization patterns have served to limit children's opportunities for IM within their neighborhoods (16, 106) and that cities are primarily designed with adults and cars in mind, not children (107). PA and active travel in rural settings, sometimes visioned as utopian environments for children to grow up in (108, 109) might also have great impact of winter darkness, where outdoor lighting is sparse or less extensive (39). Colder temperatures, wind and precipitation are presumably also contributing barriers to children's PA and active travel during winter months (38, 40). The geographical differences of PA and active travel due to daylight hours point to the need to also consider light conditions in relation to urban and rural design to understand the barriers of darkness.

Fear of darkness is common among children and influences their safety perception outdoors. This, in turn, leads to darkness constituting a barrier to CIM, both in terms of the consideration of whether to go out or not after dark as well as which places in the built environment should be avoided or not. Therefore, children have developed different coping strategies e.g., not traveling alone, avoiding certain places, or acting confident. There are apparent gender differences in the perception of safety after dark. Girls felt less safe in public spaces than boys, were more afraid of the dark and had denser walking activity spaces after dark compared to that of boys. These findings are in line with research on adults, suggesting that women tend to report more fear than men (86, 110, 111) and that insecurity and fear are factors that can limit IM (112). Also, other factors, such as the type of setting and familiarity with places, could play a role in safety perception and CIM. Hence, it seems that perceptions of safety/fear in the local environment can limit IM, but by familiarizing oneself with the area (at daytime), it could be perceived as safer (88, 113, 114). Places perceived as safe, but also offer children thing to do, can positively support CIM during dark hours (71). This is in line with research showing positive associations between place attachment (i.e., the emotional bonds between people and places) and adults' walking behaviors (115).

For older children (above 14), CIM during night-time can play a role in their identity development and transition into young adulthood, e.g., by offering avoidance of parental control, new possibilities and several “firsts” (74, 75, 82). The night can be viewed as a “second city,” with its own geography and group of people (5). Hence, the dark spaces at night might function as a refuge for older children and offer an opening “for trying to be someone the daytime may not let you be, a time for meeting people you should not, for doing things your parents told you not to do” (116). The findings highlight that promoting CIM during dark hours might not only be a contributor to the accumulation of PA, confidence, and skills, but also for their mental health. This behavior, while often negatively interpreted by adults, might fill an important function for children's self-regulation (117), pointing to the need to develop neighborhood environments in which any child can engage in play and recreational activities independently throughout the year.

The presence of outdoor lighting is related to increased PA and active travel. The perception of outdoor lighting or the lit environment can also influence activity levels, and outdoor lighting is viewed as a physical-activity-facilitator among children. However, findings are inconsistent on how and if outdoor lighting influence children's PA. The findings imply that the relationship is complex, and that child attributes, such as age, gender, and personal interests, as well as parental licenses can be mediating factors when it comes to the relations between outdoor lighting, PA and active travel.

Outdoor lighting seems to also influence children's place use, interaction with the environment and identity development. The presence and quality of outdoor lighting can influence whether children use a place, whether they perceive an area as an activity-friendly environment and affect their interactions with their surroundings and play a role in their navigation and negotiations of public space (74–78, 82). When it comes to children's route choices, however, outdoor lighting did not seem to influence children's cycling routes to an equal extent (79, 80). The reason for this is unclear and should be investigated further.

There are both structural and experienced inequalities in the spaces and places for the children's everyday outdoor life in the identified papers. Some described that there are different possibilities for children to be outdoors after dark depending on where they live (81, 83, 84) and that children experienced dimmed, broken, or absent lighting as a material manifestation of neglect from powerful institutions and class devaluation (81). In previous studies, it has been found that people living in socially vulnerable areas make fewer trips per person and per week than the national average (118, 119). It has been proposed that the way urban spaces are lighted can reinforce inequality, but also challenge them (120). Inequalities in lighting design in relation to CIM and mobility justice should further be addressed in research.

The presence of outdoor lighting and lighting quality may also play a role in children's safety perception, which in turn can influence their IM. Contrary to the literature on adult's responses to outdoor lighting these findings are still limited. Among adults, the relationship between outdoor lighting and perceived safety was by far the most frequently researched topic (121). Moreover, light sources with whiter light seem to have a positive effect on adults' safety perception (121). In our review, one paper suggested that outdoor lighting with greater green or blue hues was related to children feeling safer (96). Findings also showed that areas perceived as well-lit could be perceived as safer and that children requested brighter outdoor lighting as a way to feel safer (81, 90, 96). However, Côté-Lussier et al. (96) relied on lighting data extracted from images taken from an International Space Station rather than photometric on-site measures (e.g., illuminance, luminance, spectral power distribution) or observer-based assessments commonly used to study human perception, evaluation or behavior of the lit environment (8). The assessment of the raw image brightness values using photos from space gives a rough measure of the lighting in an area, but it is uncertain if it can be compared to the actual experience on site. Overall, the restricted number of identified papers and the limited understanding of the lit environment within these papers means that more research is needed to understand the relationship between children's safety perception and the presence and quality of outdoor lighting.

Presence of outdoor lighting might influence outdoor risks for children, but also children's perception of risks. The highest number of accidents occurred when most children were outdoors, e.g., during days with good weather, summer months, and afternoon and evening hours. Overall, the papers (e.g., 97–99) say little about the relative risk of child injuries after dark or in the presence of outdoor lighting. Only two of the papers (100, 102) showed a relationship between greater risk for child injuries and death after sunset, and that parents' perceptions of outdoor risks after dark can limit CIM. Traffic safety issues have been highlighted as a major constraint for children's active travel [e.g., (17, 122)], which emphasizes the importance of untangling the actual and perceived risks of accidents when children are independently mobile during dark hours and the potential of outdoor lighting to influence such risks.

The presence of outdoor lighting is associated with decreased odds of child homicide (101). It has been claimed that outdoor lighting can reduce crime and increase public safety (123), but it has also been disputed (124) and criticized as an approach that elude the deeper socio-economical causes of crime (125–127). The connection between outdoor lighting and crime appears to be sensitive to local conditions and should be context appropriate (128, 129). Since the findings in this review is only based on one paper, no conclusions can be made between the relationship of outdoor lighting and children's risks of being victims of crime.

It should be noted that there are legitimate differences regarding the age when children are allowed by their parents to be independently mobile (e.g., in their neighborhood or commuting), but also that children of different ages have different needs of independence. The focus in this review has been on children within school age (6–18 years old), which brings big differences in the degree of their IM. Despite this, the findings point to how darkness can be a barrier for IM for children of all ages, while also illustrating how this barrier is realized in various ways. For younger children, parental constraints regarding darkness can limit their possibilities to be outdoors and entail e.g., being driven to activities [e.g., Forsberg et al. (50), Nakanishi and Black (102)]. Older children could also be subject to parental constraints, but here it appears that darkness also entails spatial barriers, leading to parental restrictions in the form of “no-go” areas outdoors [e.g., James and Embrey (51), Pooley et al. (70)]. Overall, the findings indicate that outdoor lighting, when perceived as adequate, has an impact on CIM for all children within school age.

### 4.2. Current limitations in understanding the quality of outdoor lighting from a child perspective

In order to cover a variety of perspectives of CIM during dark hours and the role of outdoor lighting, both natural light and outdoor lighting were considered in this scoping review. In RQ 3 we asked: *How are the light conditions defined and operationalized in relation to CIM in previous studies?* with the objective to further the understanding of the light conditions *per se*.

A drawback that has emerged in the papers is the lack of details about the studied outdoor lighting. Several of the papers discuss the existence or non-existence of lighting and mention “poor lighting,” “well-lit,” “sufficient light,” “better lighting” or “good lighting” in relation to children's use and experiences of the outdoor environment, but few of them focus in detail on the qualities of outdoor lighting to meet children's needs for IM. What is meant with “well-lit” or “good lighting” from the perspective of children? What type of light source or lighting design could support CIM? Research that includes both observer-based and technical-based environmental assessments of the studied lighting could provide better insights into these matters [cf., (10, 130, 131)]. Côté-Lussier et al. (96) is the only paper that has used technical assessments of the outdoor lighting, but the assessments are arguably too simplified and rough. To better understand the associations between CIM and outdoor lighting, more in-depth research that takes both technical aspects of lighting and the lit environment, together with observer-based assessments from children's perspectives into account, is needed. A good starting point would be to base these assessments on well-established and proven methods within lighting research and child studies.

There are also limitations in the chosen methods and included perspectives in the identified papers with regard to understanding how outdoor lighting could support CIM. Since parental factors, such as parents' perceptions of the neighborhood, parental license and parents previous IM experiences, influence CIM (132), it is of relevance to understand parents' perspectives of light conditions. This is reflected in the identified papers to a limited extent, with parents' views included in 25% of the studies. Nevertheless, children's views of their environments should not be ignored. The basis that IM is every child's right and that they have the right to be heard and to express their views on matters affecting them (Article 12) (4) should be a point of departure. Children's perspectives on light conditions captured through self-reports (e.g., through questionnaires or interviews), were included in 47 of the papers, indicating that their perspectives are considered to a great extent. Additionally, the included children's attributes varied between the papers, representing children of different age groups, genders and countries. However, a predominant part of the papers was based in European or North American contexts, and focused to a great extent on urban environments, indicating that the findings are mostly based on a Western and urban perspective. To better understand CIM during dark hours, research including children from different parts of the world and different kinds of settings is desired.

When studying the methods used for children's self-reports in the papers, it is also apparent that the opportunity for children to express their views on outdoor lighting are limited. Thirty-one of the 47 papers base their findings on questionnaires, but few of these include children's perceptions of lighting or the lit environment, and if they do, the children are asked to answer a statement about how they perceive the lighting (e.g., positive or negative) or if lighting is present or not [e.g., Evenson et al. (57), Kamargianni et al. (67), Onywera et al. (68)]. Instead, most of the papers that include aspects of outdoor lighting have assessed its presence in an area through GIS or so called “objective measures” on site [e.g., Sallis et al. (54), Goon et al. (58), Edwards et al. (76), Flowers et al. (77)]. There are fewer qualitative papers on children's perspectives on outdoor lighting, but these include more detailed descriptions on how children are impacted by light conditions in their outdoor lives [e.g., Mier et al. (65), Mecca (75), Thoma et al. (81)]. Here, however, technical-based environmental assessments are lacking, making it difficult to interpret the findings. As previously concluded, technical-based environmental assessments in combination with methods capturing children's and parents' perspectives [e.g., Derr et al. (133)] could provide better insights into the matter of the effect of outdoor lighting on CIM. Detailed knowledge of the lighting would aid in the understanding of how to promote exploration, play and socializing for children in their local environments in addition to supporting their essential commuting to and from school and other educational activities.

Another drawback in the papers is that the theoretical starting points are rarely explicitly stated, making it difficult to evaluate and interpret the findings. Recent systematic reviews on CIM have shown that it is associated with several attributes, such as socio-demographic, social and physical (134–136). It is therefore motivated to use theories that enable addressing the child-environment relationship. Of the 67 identified papers, only 12 refer to such theories. To better understand the complexity of CIM in relation to light conditions, using a framework considering ways in which parent and child attributes can intersect with neighborhood characteristics, is suggested in future research.

### 4.3. Methodological reflection

The limited findings about CIM during dark hours and the role of outdoor lighting might depend on shortcomings of the eligibility criteria and search strategy used. Additional themes might have emerged if papers written in other languages than English had been included. Also, the addition of non-peer-reviewed reports could have resulted in greater insights on how different light sources and lighting design might affect children's opportunities and needs. Nevertheless, the peer-review criterion was considered as the best way to identify and map the available scientific knowledge on CIM during dark hours and to identify potential research gaps for future studies. This is motivated by the fact that few studies focus on children's needs and experiences of light conditions in their everyday outdoor life, thereby implying that outdoor lighting recommendations and standards are informed by research based on adult's perceptions.

Due to limited possibilities in conducting full-text searches in all of the selected databases, papers that include aspects of light conditions may have been missed. There are existing papers that could have been relevant to include in the review that were not incorporated since they were not identified through the search process (e.g., found through colleagues, Google searches etc.). To ensure that the findings of this review can be updated and replicated, papers identified through methods other than citation searching was excluded. Despite these limitations, we believe that the most relevant papers have been found and included, when we consider the aim of this review.

## 5. Conclusion

Both natural light and outdoor lighting seem to play an important role in children's everyday outdoor life by supporting or hindering specific behaviors and needs. Findings that increase the understanding of *the quality* of outdoor lighting to support children's opportunities to move around independently during dark hours is, however, limited. This calls for future research on how different light sources and lighting design might affect CIM during dark hours. In order to develop outdoor lighting that support both children's and parents' perspectives in urban design, more research is needed to fill the gaps:

- Taking technical aspects of outdoor lighting and the lit environment into account (e.g., through photometric on-site measures), to understand the association between CIM and physical properties as well as quality of the lighting would facilitate integration in current lighting standards for urban settings.
- Focusing on specific aspects of CIM in relation to outdoor lighting, such as outdoor risks (actual and perceived), children's safety perception and route- and transportation mode choices to school and extra-curricular activities. CIM also needs to be studied during dark hours in relation to play, exploration and socialization.
- Conducting studies among children of different ages, genders and from different countries and within different kinds of neighborhood settings.

Finally, in order to promote a lighting practice that takes a child perspective into account, research-based strategies including children's perceptions and perspectives are essential. By conducting research with children we can really understand what they think about issues that affect them (137). Our hope is that this scoping review will serve as a starting point for an attempt to create bridges between children's perspectives, urban planning and lighting practice, thereby furthering the understanding of the relationship between built environment characteristics and CIM. This could lead to securing children's perspectives in the rapid development of energy efficient outdoor lighting and creating pathways for CIM over the day and seasons.

## Data availability statement

The original contributions presented in the study are included in the article/Supplementary material, further inquiries can be directed to the corresponding author.

## Author contributions

Funding for the study was obtained by JR, PM, and MJ. AL identified and initially analyzed the papers with the support by JR, PM, and MJ, and wrote the first draft of the manuscript. JR, PM, and MJ contributed to revision. All authors contributed to conception and design of the study and contributed to the article and approved the submitted version.

## Footnotes

^1^Australia, Brazil, Denmark, England, Finland, France, Germany, Ireland, Israel, Italy, Japan, Norway, Portugal, South Africa, Sri Lanka and Sweden.

^2^Meaning that the clocks would be one hour ahead of Greenwich Mean Time (GMT) in the winter and two hours ahead of GMT in the summer.

## Supplementary material

Supplementary Table 1 Date for search and the search string used in each database.

Supplementary Table 2 List of papers included in the review. The papers are presented in chronological order of author's/s' name/s and details about how CIM has been studied is presented.
