# Supplementary material for: Children's independent mobility during dark hours: a scoping review
Source: Front Public Health. 2023 Jun 9;11:1110224. doi: 10.3389/fpubh.2023.1110224 (PMC10288107; doi:10.3389/fpubh.2023.1110224)
Supplement: Supplementary Table 1 — Date for search and the search string used in each database. [file Data_Sheet_1.PDF]

Database	Date	Search string
Scopus	April 14, 2021	TITLE-ABS-KEY(("child*" OR "infant" OR "pupil*" OR "young people" OR "youth" OR "teenage*" OR "adolescent*") AND ("mobility" OR "travel" OR "transport*" OR "play" OR "move*" OR "cycl*" OR "bicycle" OR "wayfinding" OR "by foot" OR "walk*" OR "roam" OR "access" OR "physical activity") AND ("dark*" OR "night*" OR "winter" OR "evening" OR "light*" OR "lamp*" OR "illumination" OR "lit" OR "street lighting" OR "outdoor lighting" OR "road lighting" OR "mesopic" OR "high pressure sodium" OR "LED light*" OR "metal halide" OR "brightness") AND ("outdoor*" OR "environment" OR "neighborhood" OR "neighbourhood" OR "urban" OR "city" OR "park" OR "green area" OR "green space" OR "street*" OR "residential" OR "built environment" OR "child-friendly environment" OR "public place" OR "local environment"))
Web of Science	April 19, 2021	AB=(("child*" OR "infant" OR "pupil*" OR "young people" OR "youth" OR "teenage*" OR "adolescent*") AND ("mobility" OR "travel" OR "transport*" OR "play" OR "move*" OR "cycl*" OR "bicycle" OR "wayfinding" OR "by foot" OR "walk*" OR "roam" OR "access" OR "physical activity") AND ("dark*" OR "night*" OR "winter" OR "evening" OR "light*" OR "lamp*" OR "illumination" OR "lit" OR "street lighting" OR "outdoor lighting" OR "road lighting" OR "mesopic" OR "high pressure sodium" OR "LED light*" OR "metal halide" OR "brightness") AND ("outdoor*" OR "environment" OR "neighborhood" OR "neighbourhood" OR "urban" OR "city" OR "park" OR "green area" OR "green space" OR "street*" OR "residential" OR "built environment" OR "child-friendly environment" OR "public place" OR "local environment"))
PsycInfo	April 20, 2021	AB (("child*" OR "infant" OR "pupil*" OR "young people" OR "youth" OR "teenage*" OR "adolescent*")) AND AB (("mobility" OR "travel" OR "transport*" OR "play" OR "move*" OR "cycl*" OR "bicycle" OR "wayfinding" OR "by foot" OR "walk*" OR "roam" OR "access" OR "physical activity")) AND TX (("dark*" OR "night*" OR "winter" OR "evening" OR "light*" OR "lamp*" OR "illumination" OR "lit" OR "street lighting" OR "outdoor lighting" OR "road lighting" OR "mesopic" OR "high pressure sodium" OR "LED light*" OR "metal halide" OR "brightness")) AND AB (("outdoor*" OR "environment" OR "neighborhood" OR "neighbourhood" OR "urban" OR "city" OR "park" OR "green area" OR "green space" OR "street*" OR "residential" OR "built environment" OR "child-friendly environment" OR "public place" OR "local environment"))
ERIC	April 30, 2021	AB (("child*" OR "infant" OR "pupil*" OR "young people" OR "youth" OR "teenage*" OR "adolescent*")) AND AB (("mobility" OR "travel" OR "transport*" OR "play" OR "move*" OR "cycl*" OR "bicycle" OR "wayfinding" OR "by foot" OR "walk*" OR "roam" OR "access" OR "physical activity")) AND TX (("dark*" OR "night*" OR "winter" OR "evening" OR "light*" OR "lamp*" OR "illumination" OR "lit" OR "street lighting" OR "outdoor lighting" OR "road lighting" OR "mesopic" OR "high pressure sodium" OR "LED light*" OR "metal halide" OR "brightness")) AND AB (("outdoor*" OR "environment" OR "neighborhood" OR "neighbourhood" OR "urban" OR "city" OR "park" OR "green area" OR "green space" OR "street*" OR "residential" OR "built environment" OR "child-friendly environment" OR "public place" OR "local environment"))
Engineering Village	April 30, 2021	(((((("child*" OR "infant" OR "pupil*" OR "young people" OR "youth" OR "teenage*" OR "adolescent*")) WN AB) AND ((("mobility" OR "travel" OR "transport*" OR "play" OR "move*" OR "cycl*" OR "bicycle" OR "wayfinding" OR "by foot" OR "walk*" OR "roam" OR "access" OR "physical activity")) WN ALL)) AND ((("dark*" OR "night*" OR "winter" OR "evening" OR "light*" OR "lamp*" OR "illumination" OR "lit" OR "street lighting" OR "outdoor lighting" OR "road lighting" OR "mesopic" OR "high pressure sodium" OR "LED light*" OR "metal halide" OR "brightness")) WN ALL)) AND ((("outdoor*" OR "environment" OR "neighborhood" OR "neighbourhood" OR "urban" OR "city" OR "park" OR "green area" OR "green space" OR "street*" OR "residential" OR "built environment" OR "child-friendly environment" OR "public place" OR "local environment")) WN ALL))
