# Supplementary material for: Children's independent mobility during dark hours: a scoping review
Source: Front Public Health. 2023 Jun 9;11:1110224. doi: 10.3389/fpubh.2023.1110224 (PMC10288107; doi:10.3389/fpubh.2023.1110224)
Supplement: Supplementary Table 2 — List of papers included in the review. The papers are presented in chronological order of author's/s' name/s and details about how CIM has been studied is presented. [file Data_Sheet_2.PDF]

Author/s	Year	Country	Outcome in relation to CIM	What the findings is based on	Age and gender of children	Setting	Light conditions	Theory	Method	Effect of light conditions	Theme / category
Aggio et al.	2015	England	Physical activity and sedentary time	Measurements on children	5-15, girls and boys	Urban areas	Light exposure during summer	Not defined	Quantitative (daily step counts)	Yes	Physical activity and active travel / Seasonality, day length and darkness
Bhosale et al.	2017	New Zealand	Independent mobility, unsupervised travel, active transport	Child and parent self-reports	12-14, girls and boys	Neighborhood	After dark	Not defined	Quantitative (questionnaire)	Yes	Outdoor activities and place use / After dark, Physical activity and active travel / Seasonality, day length and darkness
Carver et al.	2008 b	Australia	Physical activity (including active transport)	Child and parent self-reports	8- 9, 13-15, girls and boys	Neighborhoods with diverse road environments	After school /evenings /weekend days	Not defined	Quantitative (Child- /parent-reported, daily step counts)	Yes	Physical activity and active travel / Seasonality, day length and darkness
Carver et al.	2009	Australia	Physical activity (including active transport)	Child and parent self-reports	8-9, 13-15, girls and boys	Neighborhoods with diverse road environments	Before school/after school/ evening	Not defined	Quantitative (Child- /parent-reported, daily step counts)	No	Physical activity and active travel / Seasonality, day length and darkness
Carver et al.	2008 a	Australia	Physical activity and perception of safety	Child and parent self-reports	8-9, 13-15, girls and boys	Neighborhood	Evening, night	Not defined	Quantitative (questionnaire)	Yes	Safety perception / Dark hours and darkness
Clifton et al.	2009	US	Pedestrian-vehicle crashes	Data from database	0-15, girls and boys	Urban area	Daylight / after dark	Not defined	Quantitative (pedestrian–vehicle crash data)	Yes	Outdoor risks / Presence of outdoor lighting
Côté-Lussier et al.	2015	Canada	Perceived safety	Child and parent self-reports	8-10, girls and boys	Neighborhood	Levels of lighting	Socio-ecological model	Quantitative (questionnaire)	Yes	Safety perception / Perception of outdoor lighting
Côté-Lussier et al.	2020	Canada	Safety, health	Child and parent self-reports	8-10, girls and boys	Neighborhood	Night-time light	Not defined	Quantitative (questionnaire)	Yes	Safety perception / Perception of outdoor lighting
Crawford et al.	2008	Australia	Physical activity, play	Measurements on children	5-6, 10-12, girls and boys	Public open spaces	Presence of lighting	Not defined	Quantitative (physical activity data)	Yes	Outdoor activities and place use / Presence and quality of outdoor lighting
Culyba et al.	2016	US	Risks of homicide	Measurements on children	13-20, girls and boys	Urban area	Presence of streetlighting	Broken windows theory	Quantitative (adolescent homicide data)	Yes	Outdoor risks / Presence of outdoor lighting
Da Silva et al.	2017	Brazil	Physical activity	Child self-reports and measurements on children	0-18, girls and boys	Neighborhood	Street lighting	The ecological model	Quantitative (questionnaire, daily step counts)	Yes	Physical activity and active travel / Presence, density and perception of outdoor lighting
Dessing et al.	2016	Netherlands	Route choice, active transportation to school	Measurements on children	8-12, girls and boys	Actual and shortest walking and cycling routes to school	Streetlights	Not defined	Quantitative (GPS)	No	Outdoor activities and place use / Presence and quality of outdoor lighting

DiMaggio & Durkin	2002	US	Child pedestrian injuries	Data from database	0-20, girls and boys	Urban area	Daylight / after dark on a lighted road	Not defined	Quantitative (pedestrian–vehicle crash data)	Yes	Outdoor risks / Presence of outdoor lighting
Durkin et al.	1999	US	Child pedestrian and bicyclist injuries	Data from database	0-17, girls and boys	Urban area	Daylight / after dark	Not defined	Quantitative (pedestrian–vehicle crash data)	Yes	Outdoor risks / Presence of outdoor lighting
Edwards et al.	2015	Australia	Physical activity, park use	Child self-reports	12-15, girls and boys	Parks	Lighting around courts	Not defined	Quantitative (questionnaire)	Yes	Outdoor activities and place use / Presence and quality of outdoor lighting
Ergler et al.	2013	New Zealand	Independent outdoor play	Child and parent self-reports	8-10, girls and boys	Central city and suburban area	Winter, after dark, dark, shorter daylight hours	Bourdieu's theory of practice, affordances	Qualitative (interviews, drawing)	Not applicable	Outdoor activities and place use / After dark
Ergler et al.	2016	New Zealand	Outdoor activities, active lifestyle, outdoor play	Child and parent self-reports	8-10, girls and boys	Central city and suburban area	Winter, after dark, dark, shorter daylight hours)	Not defined	Qualitative (interviews, drawing)	Not applicable	Outdoor activities and place use / After dark
Evenson et al.	2006	US	Physical activity and active transport to school	Child self-reports	11-14, girls	Urban area	Streets are well lit at night	The socio-ecologic framework	Quantitative (questionnaire)	No	Physical activity and active travel / Presence, density and perception of outdoor lighting
Evenson et al.	2007	US	Physical activity and sedentary behavior	Child self-reports	11-12, girls	Urban area	Artificial lighting	Not defined	Quantitative (questionnaire)	Yes	Physical activity and active travel / Presence, density and perception of outdoor lighting
Evenson et al.	2010	US	Physical activity and sedentary behavior	Child self-reports and measurements on children	11-14, girls	Neighborhood	Well lit	Not defined	Quantitative (Questionnaire, daily step counts)	No	Physical activity and active travel / Presence, density and perception of outdoor lighting
Ferrao et al.	2013	Portugal	Perception of safety, child obesity	Parent self-reports and measurements on children	3-10, girls and boys	Urban area	Natural light and darkness	Not defined	Quantitative (questionnaire, anthropometric measures)	Yes	Physical activity and active travel / Seasonality, day length and darkness
Flowers et al.	2019	Australia	Physical activity, visits	Parent self-reports	3-11, girls and boys	Parks	Artificial lighting	Not defined	Quantitative (questionnaire)	Yes	Outdoor activities and place use / Presence and quality of outdoor lighting
Forsberg et al.	2020	Sweden	Active school travel	Parent self-reports	7-13, girls and boys	Rural and urban settings	Artificial lighting	The theory of planned behavior	Qualitative (interviews)	Not applicable	Physical activity and active travel / Seasonality, day length and darkness

Goodman et al.	2012	England	Physical activity and participation in out-of-home play, sports, and active travel	Child self-reports and measurements on children	8-11, girls and boys	Not defined	‘Short’ days (7.9–9.5 hours), ‘medium’ days (10.2–12.6 hours, and ‘long’ days (14.1–16.6 hours)	Not defined	Quantitative (Diaries, daily step counts)	Yes	Physical activity and active travel / Seasonality, day length and darkness
Goodman et al.	2014	Australia, Brazil, Denmark, England, Estonia, Madeira, Norway, Switzerland, US	Physical activity	Measurements on children	5-16, girls and boys	Different countries	Hour of sunset	Not defined	Quantitative (daily step counts)	Yes	Physical activity and active travel / Seasonality, day length and darkness
Goon et al.	2020	Canada	Physical activity and sedentary time	Child self-reports	9-14, girls and boys	Neighborhood	Presence of street lighting	Not defined	Quantitative (questionnaire)	No	Physical activity and active travel / Presence, density and perception of outdoor lighting
Gracia-Marco et al.	2013	Austria, Germany, Greece, Italy, France, Sweden	Physical activity and sedentary time	Measurements on children	12-17, girls and boys	Different countries	Autumn, winter, summer, spring	Not defined	Quantitative (daily step counts)	Yes	Physical activity and active travel / Seasonality, day length and darkness
Harrison et al.	2017	Australia, Belgium, Denmark, Estonia, Madeira, Norway, Switzerland, UK, US	Physical activity	Measurements on children	3-18, girls and boys	Different countries	Hours of daylight	Not defined	Quantitative (daily step counts)	Yes	Physical activity and active travel / Seasonality, day length and darkness
He et al.	2014	China	Physical activity and walkability	Child self-reports	10-11, girls and boys	Neighborhood	Sufficient lighting	Social ecological models of health behavior	Quantitative (nominal groups)	Yes	Physical activity and active travel / Presence, density and perception of outdoor lighting
Hermosillo-Gallardo et al.	2020	Mexico	Perception of safety and physical activity	Child self-reports	15, 18, girls and boys	Neighborhood	Artificial lighting	Not defined	Quantitative (questionnaire)	Yes	Physical activity and active travel / Presence, density and perception of outdoor lighting
Hidding et al.	2018	The Netherlands	Physical activity, activity friendliness	Child self-reports	13-15, girls and boys	Not defined	Proper/good lighting	Not defined	Mixed (concept mapping)	Yes	Outdoor activities and place use / Presence and quality of outdoor lighting

Jago et al.	2005	US	Physical activity	Measurements on children	10-14, boys	Urban area	Presence of streetlights	Not defined	Quantitative (daily step counts)	Yes	Physical activity and active travel / Presence, proportion and perception of outdoor lighting
James & Embrey	2001	Australia	Choice of active or passive leisure activities	Child self-reports	15, girls	Recreational spaces	Darkness / adequate lighting	Not defined	Quantitative (questionnaire)	Yes	Physical activity and active travel / Seasonality, day length and darkness, Outdoor activities and place use / After dark, Safety perception / Dark hours and darkness
Johansson et al.	2009	Sweden	Perception of fear, parental licensing	Child self-reports	13-14, girls and boys	Neighborhood	Darkness, evening	Not defined	Quantitative (questionnaire)	Yes	Safety perception / Dark hours and darkness
Johansson et al.	2010	Sweden	Independent mobility, transport to school	Child self-reports	13-14, girls and boys	Neighborhood	Darkness, evening	Not defined	Quantitative (questionnaire)	Yes	Safety perception / Darkness conditions after dark
Johansson et al.	2012	Sweden	Threats when moving in public space	Child self-reports	15-16, girls and boys	Outdoor public places	Dark, evening, night	Not defined	Qualitative (focus groups)	Not applicable	Safety perception / Darkness conditions after dark
Jones et al.	2000	England	Access and independent mobility	Child self-reports	13-14, girls and boys	Urban, suburban and rural areas	After dark, evening, night	Social model	Mixed (questionnaire, focus groups)	Yes	Safety perception / Dark hours and darkness
Kamargianni & Polydoropoulou	2014	Greece	Walking to school and walkability	Child self-reports	12, 18, girls and boys	Urban, rural and insular area	Poor lighting	Not defined	Quantitative (questionnaire)	Yes	Physical activity and active travel / Presence, density and perception of outdoor lighting
Khawaja et al.	2020	England	MVPA levels	Child self-reports and measurements on children	9-13, girls and boys	Not defined	Autumn, winter, summer	Not defined	Mixed (global positioning systems (GPS) and heart rate (HR) monitor, focus groups)	Yes	Physical activity and active travel / Seasonality, day length and darkness
Larouche et al.	2019	Canada	School travel mode and MVPA	Child self-reports	9-10, girls and boys	Urban and rural areas	Autumn, winter, spring	Not defined	Quantitative (child-reports)	Yes	Physical activity and active travel / Seasonality, day length and darkness
Lopes et al.	2014	Portugal	Children's autonomy, independent mobility, active travel	Child and parent self-reports	10-18, girls and boys	Inner city, urban, suburban, small town and rural areas	After dark	Not defined	Quantitative (questionnaire)	Yes	Outdoor activities and place use / Presence and quality of outdoor lighting
Loucaides et al.	2004	Cyprus	Physical activity level	Parent self-reports and measurements on children	11-12, girls and boys	Urban and rural areas	Winter / Summer (2 hours daylight difference)	Not defined	Quantitative (Questionnaire, daily step counts)	Yes	Physical activity and active travel / Seasonality, day length and darkness

McCray & Mora	2011	US	Perceived safety	Child self-reports	14-18, girls and boys	Activity spaces	Night, night-time	Not defined	Mixed (mapping exercise)	Yes	Safety perception / Dark hours and darkness, Perception of outdoor lighting
McCrorie et al.	2020	Scotland	Physical activity and sedentary levels, walkability index	Measurements on children	10-11, girls and boys	Urban and rural areas	Autumn, winter, summer, spring	The socio-ecological model	Quantitative (daily step counts)	Yes	Physical activity and active travel / Seasonality, day length and darkness
Mecca	2019	Spain	Nightlife leisure	Child self-reports	14-16, girls and boys	Neighborhood/ nightscape	Evening, night-time, dark	Cultural geographical framework	Qualitative (interviews, focus groups, participatory mapping)	Not applicable	Outdoor activities and place use / After dark, Presence and quality of outdoor lighting
Mecca	2020	Spain	Nightlife leisure	Child self-reports	14-16, girls and boys	Neighborhood/ nightscape	Evening, night-time, dark	Cultural geographical framework	Qualitative (interviews, focus groups, participatory mapping)	Not applicable	Outdoor activities and place use / Presence and quality of outdoor lighting, Safety perception / Dark hours and darkness
Mier et al.	2013	US	Physical activity	Child self-reports	8-13, girls and boys	Underserved neighborhoods	Dark streets/ no lights on the streets)	Not defined	Qualitative (focus groups)	Not applicable	Physical activity and active travel / Presence, density and perception of outdoor lighting
Mullan	2003	Wales	Perceptions of safety, friendliness, appearance, play facilities, helpfulness of people	Child self-reports	11-16, girls and boys	Neighborhood	After dark	Not defined	Quantitative (questionnaire)	Yes	Safety perception / Dark hours and darkness
Nakanishi & Black	2016	Japan	Displacement and issues of transport to school	Child and parent self-reports	1-18, girls and boys	Post-disaster community	Lack of streetlights	Not defined	Qualitative (interviews)	Not applicable	Outdoor risks / Presence of outdoor lighting
Nance et al.	2004	US	Motor vehicle crashes involving child pedestrians	Data from database	0-18, girls and boys	Urban area	Daylight / dark with lights / dark with no light	Not defined	Quantitative (pedestrian–vehicle crash data)	Yes	Outdoor risks / Presence of outdoor lighting
Nelson & Woods	2010	Ireland	Active commuting to school	Child self-reports	15-16, girls and boys	Neighborhood	Well-lit streets	Social-ecological theory	Quantitative (questionnaire)	Yes	Physical activity and active travel / Presence, density and perception of outdoor lighting
Nguyen et al.	2021	Australia	Time allocation	Child and parent self-reports	0-15, girls and boys	Residential location	Hours of daylight	Not defined	Quantitative (questionnaire, interviews, time-use diaries)	Yes	Outdoor activities and place use / After dark
Onywera et al.	2018	Kenya, Mozam-bique, and Nigeria	Active transportation	Child and parent self-reports	10-12, girls and boys	Neighborhood	Not good lighting	Not defined	Quantitative (questionnaire)	Yes	Physical activity and active travel / Presence, density and perception of outdoor lighting

Pizarro et al.	2012	Portugal	Physical activity and active transport	Child self-reports	15-17, girls and boys	Neighborhood	Streets well-lit at night	Not defined	Quantitative (questionnaire)	Yes	Physical activity and active travel / Presence, density and perception of outdoor lighting
Pooley et al.	2010	England	Travel, engagement with the environment	Child self-reports	12-14, girls and boys	School way	After dark, evening, night	Not defined	Mixed (GPS, photographs, texts)	Yes	Outdoor activities and place use / After dark
Quante et al.	2019	US	Physical activity	Measurements on children	12-14, girls and boys	Not defined	Autumn, winter, summer, spring)	Not defined	Quantitative (daily step counts)	Yes	Physical activity and active travel / Seasonality, day length and darkness
Reis et al.	2009	Brazil	Physical activity practices	Child self-reports	15-17, girls and boys	Parks	Artificial lighting	Not defined	Quantitative (questionnaire)	Yes	Physical activity and active travel / Presence, density and perception of outdoor lighting
Risova	2021	Slovakia	Walking, walking activity space	Child self-reports	13-16, girls and boys	City center	Night, after dark, darkness, insufficient/lack of lighting	Not defined	Mixed (mapping activity)	Yes	Safety perception / Dark hours and darkness
Risova & Madajova	2020	Slovakia	Perceived safety, walkability	Child self-reports	13-16, girls and boys	City center	Night, after dark, darkness, insufficient/lack of lighting	Not defined	Mixed (mapping activity)	Yes	Safety perception / Dark hours and darkness
Robinson	2009	England	Leisure, transition	Child self-reports	15-16, girls and boys	Nightscapes, leisure spaces	Dark, after dark	Cultural geographical framework	Qualitative (interviews)	Not applicable	Outdoor activities and place use / Presence and quality of outdoor lighting
Sallis et al.	2015	US	Physical activity	Child self-reports and measurements on children	6-16, girls and boys	Neighborhood	Artificial lighting	Not defined	Quantitative (questionnaire, daily step counts)	Yes	Physical activity and active travel / Presence, density and perception of outdoor lighting
Thomas et al.	2018	UK	Sense of place, identity, interaction with surroundings	Child self-reports	14-15, girls and boys	City	Darkness, night, broken and/or absent streetlights	Cultural geographical framework	Qualitative (interviews)	Not applicable	Outdoor activities and place use / Presence and quality of outdoor lighting, Safety perception / Perception of outdoor lighting
Timperio et al.	2008	Australia	MVPA levels	Measurements on children	8-9, 13-15, girls and boys	Public open spaces	Lighting along paths	The social-ecological model	Quantitative (daily step counts)	No	Physical activity and active travel / Presence, density and perception of outdoor lighting
Verhoeven et al.	2018	Belgium	Active transport, cycling, route choice	Measurements on children	13-15, girls and boys	Actual and shortest cycling routes	Artificial lighting	Not defined	Quantitative (GPS)	No	Outdoor activities and place use / Presence and quality of outdoor lighting

Wiebe et al.	2014	US	Perceived safety while in different transportation modes	Child self-reports	10-18, girls and boys	Transit environments	Dark, night, night-time	Not defined	Quantitative (questionnaire, activity report)	Yes	Safety perception / Dark hours and darkness
Wilson et al.	2011	US	Physical activity	Child self-reports and measurements on children	11-12, girls and boys	Neighborhood	Presence of lighting	Ecologic model	Quantitative (questionnaire, BMI, daily step counts)	Yes	Physical activity and active travel / Presence, density and perception of outdoor lighting
Yang et al.	2011	US	Active transportation (walking and bicycling)	Child and parent self-reports	5-17, girls and boys	Different urbanization levels	Summer/ winter	Not defined	Quantitative (Child-/parent-reported, active transportation data)	Yes	Physical activity and active travel / Seasonality, day length and darkness
